# Understanding the behavioural determinants of seeking and engaging in care for knee osteoarthritis in persons with type 2 diabetes mellitus: A qualitative study using the theoretical domains framework

**DOI:** 10.1016/j.ocarto.2022.100305

**Published:** 2022-08-12

**Authors:** Lauren K. King, Owen Krystia, Esther J. Waugh, Crystal MacKay, Ian Stanaitis, Jane Stretton, Alanna Weisman, Noah M. Ivers, Janet A. Parsons, Lorraine Lipscombe, Gillian A. Hawker

**Affiliations:** a Department of Medicine, University of Toronto, Toronto, ON, Canada; b Women's College Research Institute, Women's College Hospital, Toronto, ON, Canada; c Department of Physical Therapy, University of Toronto, Toronto, ON, Canada; d Patient Research Partner, Toronto, ON, Canada; e Lunenfeld-Tanenbaum Research Institute, Mount Sinai Hospital, Toronto, ON, Canada; f Department of Community and Family Medicine, University of Toronto, Toronto, ON, Canada; g Applied Health Research Centre and Li Ka Shing Knowledge Institute, St. Michael's Hospital – Unity Health Toronto, Toronto, ON, Canada

**Keywords:** Osteoarthritis, Type 2 diabetes mellitus, Qualitative, Theoretical domains framework

## Abstract

**Objectives:**

Symptomatic knee osteoarthritis (OA) frequently co-occurs in individuals with type 2 diabetes mellitus (T2DM). In the context of T2DM, OA is often underdiagnosed and undertreated. To elucidate strategies to improve OA care in persons with T2DM, we assessed their perceptions of the barriers and enablers to seeking and engaging in OA care.

**Design:**

We conducted semi-structured interviews with 18 individuals with T2DM and symptomatic knee OA in Ontario, Canada. Transcripts were deductively coded using the Theoretical Domains Framework (TDF), an implementation science framework that incorporates theoretical domains of behaviour determinants, which can be linked to behaviour change techniques. Within each of the relevant domains, data were thematically analyzed to generate belief statements.

**Results:**

Seven of the TDF domains prominently influenced the behaviour to seek and engage in OA care. Participants described insufficient receipt of OA knowledge to fully engage in care (knowledge), feeling incapable of participating in physical activity due to joint pain (beliefs about capabilities), uncertainty about effectiveness of therapies (optimism) and lack of guidance from health care providers and insufficient access to community programs/supports (environmental context and resources). Key enablers were strong social support (social influences), sources of accountability (behavioural regulation) and experiencing benefit from treatment (reinforcement). Participants did not see concomitant T2DM as limiting the desire to seek OA care.

**Conclusions:**

Among individuals with symptomatic knee OA and T2DM, we identified behavioural determinants of seeking and engaging in OA care. These will be mapped to behavioural change techniques to inform development of a complex intervention.

## 1 Introduction

The number of persons living with type 2 diabetes mellitus (T2DM) is rapidly rising [1] with substantial impact on the lives of those affected, including a greater than two-fold increase in risk of all-cause death [2]. Approximately 30–50% of persons living with T2DM also have osteoarthritis (OA) [3,4]. The frequent clustering of T2DM and OA [5,6] driven by shared risk factors, such as obesity and older age [7], poses a serious health burden. Painful knee OA, which accounts for nearly four fifths of the burden of OA globally [1], is an important cause of walking limitation [8] and, without appropriate care, may limit individuals with T2DM from engaging in guideline-recommended physical activity [[9], [10], [11]]. However, both health care providers and persons with OA and T2DM may focus less attention on the diagnosis and management of OA due to competing demands of the more complex disease [12]. Together these gaps lead to worse clinical outcomes, with evidence of increased risk of cardiovascular events and other diabetes-specific complications in persons with T2DM with knee OA-related walking difficulty [13]. First line nonpharmacologic self-management approaches, including physical activity/exercise that strengthen muscles around the joint to improve pain and promote physical functioning and appropriate disease education, are frequently under-utilized [14,15]. Thus, there is a critical need to elucidate strategies to improve uptake of evidence-based self-management for symptomatic knee OA in persons with T2DM. Closing this knowledge-practice gap has the potential to improve both OA and T2DM outcomes.

Complex implementation interventions, containing several interacting components, are often required to address health care challenges [16]. The overarching aim of our research is to develop a complex intervention to improve diagnosis and evidence-based management of knee OA in people with T2DM. The UK Medical Research Council (MRC) guidance on the development of complex interventions recommends, as a first step in intervention development, the use of theory to understand the determinants of the health behaviour(s) [16,17], which can involve multiple health care levels. The Theoretical Domains Framework (TDF) is an implementation science framework that incorporates a range of theoretical constructs to comprehensively identify determinants of behaviour. TDF domains can be mapped systematically to behaviour change techniques [18,19] to maximize intervention effectiveness. Further, input from multiple stakeholders throughout the intervention development process can increase impact and sustainability [20].

In the first phase of developing a theory-informed implementation intervention to improve diagnosis and treatment of knee OA in people with T2DM, we sought to understand the determinants of behaviour of key stakeholders at multiple health care levels. The objective of the current study was to comprehensively identify perceived barriers and enablers to seeking and engaging in OA care in individuals living with both T2DM and knee OA using the TDF.

## 2 Methods

### 2.1 Design

In this qualitative description study [21], we conducted semi-structured interviews with a sample of individuals with a physician diagnosis of both knee OA and T2DM living in Ontario, Canada. Interviews were completed in September and October 2020. We followed guidance by Atkins et al. [19] on the collection and analysis of qualitative data using the TDF: incorporating TDF domains in the interview guide; data coding to the TDF domains; generating themes/belief statements within each domain relevant to the behaviour of interest; and presenting findings framed by the TDF. This study was completed alongside others targeting different stakeholder groups, which will be published separately.

This study was approved by the Research Ethics Review Boards at Women's College Hospital and at the University of Toronto. We followed the Consolidated Criteria for Reporting Qualitative Studies (COREQ) guidelines for reporting qualitative research [22].

### 2.2 Study setting

In Ontario, Canada, individuals with chronic conditions, such as T2DM, present to primary care providers (family physicians or nurse practitioners) as the first point of contact in the health care system. A referral from a primary care provider or other physician is needed for an individual to access medical specialist services. The health care system in Ontario is publicly funded and privately administered. The Ontario Health Insurance Plan provides coverage for most medical and emergency services provided in Ontario. However, it does not provide universal coverage; relevant to persons with T2DM and OA, prescription drugs and physiotherapy for those who are not on social assistance and/or under age 65 are paid for out-of-pocket by patients.

### 2.3 Sampling

We recruited participants though the Arthritis Society Arthritis Rehabilitation and Education Program (AREP) in Ontario, Canada, and through the family medicine clinic at Women's College Hospital, an academic hospital in Toronto, Canada. AREP, which is funded by the provincial government, provides group arthritis education classes as well as individual assessments by arthritis therapists (physical therapists and/or occupational therapists), at no out-of-pocket cost to patients. By including participants who had accessed AREP, this allowed insight to potential enablers to care, while those recruited from family medicine could provide insight on barriers.

Individuals aged 45 years or older and who self-reported a physician diagnosis of both T2DM and knee OA were invited to participate. Emails detailing the study and eligibility criteria were sent to a mailing list of clients with osteoarthritis that had previously participated in the AREP program in the last year with details on how to contact the study team. Further, after screening electronic medical records, potentially eligible participants were approached by phone or e-mail about participating in the study. A purposive sampling strategy was employed to ensure results reflected those from a range of ages and disease duration, both of T2DM and knee OA. We stopped recruitment when additional interviews yielded no new concepts related to our research question [23], as determined by the analytic team.

### 2.4 Data collection

We developed a semi-structured interview guide that aimed to comprehensively assess determinants of behaviour according to the TDF, a validated framework structure [24]. The behaviour of interest was seeking and engaging in knee OA care in persons with both T2DM and knee OA.

At the end of each interview, we collected sociodemographic information (age range, gender, residence [urban, suburban, rural], years since diagnosis of T2DM and knee OA, and knee OA symptom severity (Western Ontario and McMaster Universities Osteoarthritis Index [WOMAC] pain subscale [25], Heath Assessment Questionnaire [HAQ] walking item [26]).

Interviews were conducted by telephone, by one of two researchers (EW or LK). Interviews lasted 30–50 ​min. Interviews were audio recorded, transcribed verbatim and anonymized prior to analysis. Data were organized using NVivo 12 software (QSR International Pty Ltd).

### 2.5 Data analysis

We first analyzed interviews using content analysis [27], deductively coding interview data to the TDF domains [19]. Two members of the research team (OK, LK) initially coded three transcripts independently to identify relevant TDF domains. Coding disagreements were resolved through discussion and review of original transcripts. Once there was agreement with how to apply codes, one researcher (OK) coded all subsequent transcripts.

Authors also remained open to inductively coding data and developing themes outside of TDF if data did not fit into a specific TDF domain, to ensure no behaviour determinants or other themes relating to our research question were missed [28]. Since we sought to understand determinants of seeking and engaging in OA care in persons with T2DM, we specifically were interested in perspectives of participants on any barriers or enablers presented by their T2DM.

We then thematically analyzed [29] data coded within each TDF domain, to inductively develop themes in the form of belief statements [19]. Thus, the belief statement(s) within each TDF domain reflected the specific determinants of seeking and/or engaging in OA care that we identified as prominent in our data. Belief statements were developed by two researchers (OK, LK) and cross-referenced with the analytic team (EW, CM) as well as a senior mixed methods researcher (GH). In addition to following guidance by Atkins et al. [19], rigour was enhanced through use of multiple analysts, analytic memos, and an audit trail [30].

## 3 Results

We interviewed 18 persons with T2DM and knee OA. Of these, 9 were women (50%), 9 were over the age of 70 years (50%), 3 were from a rural location (16.7%), 9 had lived with their diabetes diagnosis for greater than 10 years (50%), 8 had lived with an OA diagnosis for greater than 20 years (44.4%), and 13 had at least some difficulty walking outdoors on flat ground (65%). Mean WOMAC pain was 8.17/20 (SD 4.84), with a range of 2–20 (Table 1). Participants recruited from the Arthritis Society (n ​= ​14) are denoted as “PAREP” and from family medicine (n ​= ​4) as “PEFP”.

**Table 1:** Participant characteristics (n ​= ​18).

Characteristic	n (%)
Gender, woman	9 (50.0)
Age	
40–49 years	2 (11.1)
50–59 years	2 (11.1)
60–69 years	5 (27.8)
≥70 years	9 (50.0)
Residence	
Urban	12 (66.7)
Suburban	4 (22.2)
Rural	2 (11.1)
Years since diabetes diagnosis	
0-9	9 (50.0)
10-19	5 (27.8)
≥20	4 (22.2)
Years since osteoarthritis diagnosis	
0-9	5 (27.8)
10-19	5 (27.8)
≥20	8 (44.4)
Self-reported ability to walk outdoors on flat ground?	
Without any difficulty	5 (27.8)
With some difficulty	10 (55.6)
With much difficulty	2 (11.1)
Unable to do	1 (5.6)
Knee osteoarthritis pain, WOMAC pain subscale (0-20), mean (SD)	8.17 (4.84)
Recruitment	
AREP^a^	14 (77.8)
Family medicine	4 (22.2)

a AREP, Arthritis Society Arthritis Rehabilitation and Education Program.

We identified seven TDF domains as relevant to the behaviour of seeking and/or engaging in OA care and present inductively developed belief statement(s) for these domains (Table 2).

**Table 2:** Relevant Theoretical Domains Framework domains with inductively developed belief statements and illustrative quotes.

TDF Domain	Belief statements	Sample illustrative quote
**Knowledge**	When I have a strong understanding about OA, I am better able to engage in its management	*“I really would have preferred it if at the beginning when you have a problem and the doctor knows, okay, it's knee OA, we need to work a program where you strengthen your muscles, support your knee, and if you don't do this, eventually, even estimate how many years, you may need surgery. I would much prefer it because having this vague talk about the subject does not give people the importance of behaving a certain way …” PEFP-102*
**Beliefs about capabilities**	Joint pain stops me from engaging in exercise for OA treatment	*“I'm* unable *to do anything now.” PAREP-406*
**Optimism**	I do not expect that OA treatments will significantly help my symptoms	*“I think I'm just to the point where I'm almost beyond hope.” PAREP-414*
**Reinforcement**	I continue to engage with OA treatments if they improve my OA symptoms	*“It seems like if I do the exercise then the joints will not be as stiff if I'm not doing the exercise. If I miss one day of exercise, I feel the joints are very stiff and very painful. That's why it's kind of a push that I have to do exercise every morning, no matter how tired I will be.” PEFP-104*
**Environmental context and resources**	Insufficient health care provider support limits my uptake of and engagement in OA care	*“He [family doctor] focuses on my diabetes; arthritis, he just refers me to the Arthritis* Association*.” PAREP-402*
	Access to relevant facilities, programs, or resources enables management of my OA	*“I'm* not *covered, insurance-wise, so I couldn't go for physio and things like that” PAREP-404*
**Social influences**	Having social supports help me to stay engaged with OA treatments	*“My wife is the go-getter here. She would say,* let's *go down and give it a whirl and try [pool exercise] out, so we did. I don't know how she thought of it, but we just did and it seemed to work.” PEFP-101*
	I listen to peers about how to manage OA	*“And you learn more from other people. You learn how to cope with other people. And that's what I like about group settings. You talk about, oh, I can't do that because of this. And then somebody will say, oh, well you can do this, this is how I do it.” PAREP-409*
**Behavioural regulation**	I benefit from having accountability to stay engaged with my OA treatments	*“Somebody to hold you accountable, somebody to say hey did you go out and run today or, why didn't you and is there anything we need to talk about. Sort of like a sponsor in AA, somebody you could touch base with. Though I think your spouse and that can do it, I think it's always better with somebody who is going through the same thing because they know what you're experiencing.” PAREP-407*

### 3.1 Domain: knowledge

Belief statement: When I have a strong understanding about OA, I am better able to engage in its management.

Many participants described initially dismissing their OA-related symptoms, perceiving these to be a “normal” part of ageing. Only when symptoms became intolerable was treatment-seeking behaviour activated. Thus, having insufficient understanding about OA onset was a barrier to seeking early OA care.

*“I guess [I ignored my pain] because I have a high pain threshold, I was, yeah, it’s just another ache and pain growing old.” PEFP-103*

Participants spoke about the importance of having the knowledge of what their diagnosis was, i.e., receiving a diagnosis of OA. When they perceived they lacked a diagnosis for their joint symptoms during their care journey, either through insufficient attention or communication by health care providers, participants were less likely to engage in care. Once a diagnosis was made by a health care provider, insufficient communication and resultant poor understanding of OA management limited their ability to fully engage in their OA treatment.

*“I guess the education point, but then I felt like I was left with okay, now what do I do. I don’t know what to do about it.” PAREP-411*

### 3.2 Domain: beliefs about capabilities

#### 3.2.1 Belief statement: joint pain stops me from engaging in exercise for OA treatment

Participants described how OA-related joint pain was a major limiting factor stopping them from engaging in physical activity/exercise for OA treatment. Participants perceived the severity of pain made it too challenging for them to use the joint.

*“I just couldn’t exercise; I was in a tremendous amount of pain” PAREP-402*

Participants’ beliefs about OA shaped their sense of capability. For example, some perceived they could not adequately engage in physical activity due to advanced OA-related structural changes.

*“But in the end, it’s bone on bone, so there’s nothing to do except not move the bones.” PAREP-408*

### 3.3 Domain: optimism

#### 3.3.1 Belief statement: I do not expect that OA treatments will significantly help my symptoms

Many participants described a negative outlook and lack of optimism towards OA treatments. Many perceived that available OA treatments, particularly physical activity and exercise, would not significantly help their symptoms or disease trajectory, therefore acting as a barrier to their use. Some participants described how they perceived only surgical intervention would help improve their symptom state.

*“I mean, I'm not expecting utopia, I'm not expecting to have the same mobility and lack of pain that I had when I was in my teen years or 20s.” PAREP-413*

### 3.4 Domain: reinforcement

#### 3.4.1 Belief statement: I continue to engage with OA treatments if I feel they improve my OA symptoms

Participants discussed that they were more likely to continue to use an OA treatment when they perceived an improvement in their OA symptom state; otherwise, most would be likely to give it up.

“*The other thing I do which I think is beneficial is, at night, I have a heating pad … I turn off the lights and lying down, I put the heating pads between both knees. That’s glorious. The knees seem to appreciate it. I look forward to it each night.” PEFP-101*

Participants described the importance of the positive feedback that it was helping, reinforcing its use, which applied to a range of treatments, including physical activity/exercise and to passive therapies. Reinforcement could come internally, from their own experiences, or externally, such as from a health care provider indicating that a treatment was having benefit.

*“I feel much better with them [physiotherapist], and I’m sure that it’s helping me in many ways. She’s always encouraging me, she’s very glad I’m doing these exercises …” PAREP-410*

### 3.5 Domain: environmental context and resources

#### 3.5.1 Belief statement: insufficient health care provider support limits my uptake of and engagement in OA care

Participants described insufficient support from their physicians with respect to both assessment and treatment of OA. Some participants mentioned that their physicians dismissed their symptoms as a consequence of ageing, delaying their diagnosis of OA. Other participants spoke about how their physicians provided little direction for treatment of their OA, often with the focus being mostly on their T2DM. Thus, T2DM was prioritized by physicians at the expense of care for their OA.

*“[Health care providers have been] focused on the diabetes.” PAREP-412*

*“I don’t think [my family doctor] knew what to do, I think she did some simple examinations and checked to see if my hips were involved and I don’t think she knew what else to do … The osteoarthritis, I don’t think she had a clue.” PAREP-405*

*“I think the problem is a lot of people when they age, and they get aches and pains, and they're told [by health care providers], oh, it’s part of aging, be more active.” PEFP-102*

#### 3.5.2 Belief statement: access to relevant facilities, programs or resources enables management of my OA

Many participants described the importance of having access to facilities, programs, or resources to enable them to effectively manage their OA. Several participants saw their lack of private insurance coverage for physiotherapy and other allied health support as a critical barrier to accessing OA care.

*“I’m poor. I can’t. I have zero income.” PAREP-406*

Many participants spoke about how inadequate access to exercise equipment, swimming pool or other facilities also prevented them from engaging in their OA care as much as they would like. For many, it was the cost that was prohibitive. Conversely, some participants having had access, spoke about how it was critical for keeping up with their OA care.

*“At Parks and Rec, they have a heated pool, so I was going there two and three times a week, basically walking back and forth in the water because the warm water and walking I found it helpful.” PEFP-103*

### 3.6 Domain: social influences

#### 3.6.1 Belief statement: having social supports helps me stay engaged with OA treatments

Participants saw social supports, that could come in different forms, as an important enabler to engaging in their OA treatments. One participant discussed how his spouse was an important ally who encouraged him to engage with therapeutic exercise. For another participant, when his spouse became injured, his therapeutic exercise for OA decreased as he no longer had a walking companion. Lack of social support was seen as a barrier to staying engaged in OA care, as they felt unsupported and alone in treating their OA.

“*No, I’m alone. And I said, the loneliness is the mother of all evil that I’m alone.” PAREP-406*

#### 3.6.2 Belief statement: I listen to peers about how to manage OA

Participants described how they were more likely to engage in an OA treatment if it had been recommended by a peer, irrespective of whether the treatment was based on scientific literature. Sharing of ideas that enabled engagement in OA care could occur in social groups or in unmoderated internet forums. Participants reported an interest in learning about the experience with others and trying what had worked for someone else.

*“I started taking turmeric, whether that’s helped it or if that’s just psychological, I don’t know, but somebody say, try turmeric, it helps. So, I’ve been doing that for a few months.” PEFP-103*

### 3.7 Domain: behavioural regulation

#### 3.7.1 Belief statement: I benefit from having accountability to stay engaged with my OA treatments

Participants spoke about how having individuals checking in on them was important to keep them “accountable” and engaged in OA care, including attending appointments, such as for physiotherapy, or in engaging in self-management strategies, such as a physical activity routine. Participants highlighted that this source of accountability did not necessarily need to come from a healthcare provider. Participants suggested that peer support from individuals experiencing similar symptoms would be helpful due to the shared experience.

***“****I think if I ⋯ I think, sometimes, when you have a person with you to motivate you, in terms … if you are going to a physio session and that, you kind of keep … you know you have an appointment. You end up going.” PAREP-404*

### 3.8 Inductively developed theme falling outside of TDF

#### 3.8.1 Persons with diabetes want care for their OA

Participants expressed desire for treatment for their knee OA because it impacted many aspects of daily life. The presence of co-existing T2DM itself was not seen as limiting their desire for OA care.

*“I would love to be able to do more. I would love to be able to wake up in the morning and not have to scream, trying to get my legs to move. I would love to just be able to wake up and go. I had to retire at 62½ because I couldn’t do my job.” PAREP-414*

However, many participants reported that their T2DM health care providers typically did not discuss joint pain with them.

*“I do go to a diabetes clinic, where I have a diabetes doctor and he’s checking everything. They don’t talk about it [arthritis] much really. It’s more monitoring the situation, monitoring your blood sugar levels and so on.” PEFP-101*

## 4 Discussion

This qualitative study used the TDF to comprehensively explore determinants of seeking and engaging in OA care from the perspectives of persons living with knee OA and T2DM, a population where undertreatment of OA may have particularly deleterious effects. We identified important barriers and enablers across seven TDF domains. Barriers included insufficient OA knowledge to fully engage in care (knowledge), feeling incapable of participating in physical activity due to joint pain (beliefs about capabilities), low confidence that treatment would be helpful (optimism), and lack of guidance from health care providers and insufficient access to community programs/supports (environmental context and resources). Key enablers were receipt of feedback that treatment was helping (reinforcement), strong social support (social influences) and sources of accountability (behavioural regulation). While we specifically sought to understand determinants of behaviour in the context of living with another complex chronic disease, T2DM was not seen to impact participants’ desires to seek and/or engage in OA. These results, together with findings from interviews of other stakeholders, will serve as important inputs in developing a complex intervention to improve diagnosis and evidence-based treatment of OA in persons with T2DM.

Optimal management of knee OA requires that patients actively participate in care. A systematic review of patients' perceived health information needs found that patients desired more information about diagnosis, prognosis, management and prevention, and for communication to be given clearly [31]. The current study confirms how lack of information provided to patients by health care providers limits individuals' full engagement in OA care. Thus, providing adequate assessment of joint pain, making a diagnosis, and provision of information about OA should be a priority in OA care by HCPs. This may also bolster both low optimism and belief in capabilities, further barriers described by participants. It is incumbent upon health care providers to enhance patient communication, as patients’ perceptions of their disease and treatments have important implications for how they engage in care [32].

Evidence-based recommendations for management of knee OA have existed for many years [33,34], yet participants described challenges in accessing adequate care, perceiving a strong influence from the environmental context and availability of resources. Participants’ perceptions of insufficient attention to OA by their health care providers matches studies of primary care clinicians who indeed generally viewed OA as a low priority [35]. Further, participants saw insufficient government funding of physiotherapy in Canada as a potential modifiable barrier to engagement in OA care. Thus, changes to the health system are critically needed to help individuals with OA effectively engage in care to mitigate downstream consequences of undertreated OA. These include better knowledge dissemination, enhanced resource navigation to link patients with available health care and community resources for OA (for example, in Ontario AREP is government funded), and greater leverage of expertise of allied health providers for patients with complex multimorbidity.

In care for chronic conditions such as OA, making social supports available or helping individuals use the social supports available to them may help promote engagement. Participants described the importance of having others around to support their health behaviours, which included OA management. Other qualitative work has shown social influences to be an important determinant of adherence to physiotherapist-prescribed physical activity for OA [36]. Harnessing peer support, such as peer support groups and/or peer health ambassadors, may be an effective additional strategy to improve complex health behaviours required in disease management [37].

Multiple prior studies describe the “work” of living with and self-managing T2DM [38] and thus we theorized that this might lower the motivation of persons with T2DM and knee OA to seek care for their OA. We found that participants themselves wanted care, and, often needed to “work” to find it on their own. Prior qualitative research has found that OA “doesn't make it to the top of the list” [12] for primary care clinicians treating individuals with complex chronic conditions such as T2DM. This study demonstrates this view is not shared by persons living with knee OA and T2DM and highlights a potential discrepancy in health priorities between individuals with T2DM and knee OA and their health care providers.

Our study has many strengths. We used the TDF, which synthesizes a wide range of psychological theories related to behaviour change [18], to comprehensively assess determinants of behaviour, which makes it less likely we failed to capture an important barrier or enabler to seeking and engaging in OA care in persons with knee OA and T2DM. Applying the TDF also provided a well-developed theoretical underpinning and a common language, with ability to make evidence-based links to behaviour change techniques [39] in next phases of complex intervention development. Further, this is the first study to our knowledge to specifically assess barriers and enablers in the growing population with OA and other complex chronic conditions, an important patient context, and also where the ramifications of insufficient care may be greater. Other research teams may benefit from this systematic understanding when planning interventions in persons with OA.

Our study has limitations. Compared to other studies in which the TDF is applied, but fitting with our research question, our behaviour of interest (seeking and engaging in OA care) lacked specificity and some important nuances to aspects of seeking and engaging in care may have been missed. The TDF was designed for ascertaining barriers and enablers to health care behaviours in implementation research [18] and mostly has been applied to health care providers [24]. While it has been applied to the understanding of patient behaviours [40], some elements of the TDF may be less applicable. All participants had an established diagnosis of OA and most were past clients of Arthritis Society Arthritis Education and Rehabilitation Program and thus we may have missed distinct views of individuals who had not accessed any care. Other factors, such as education or race/ethnicity, may influence care seeking and we did not sample on these in the current study.

Persons with knee OA and T2DM have a complex constellation of conditions that interact and this study identified behavioural determinants of seeking and engaging in OA care from the perspectives of persons living with knee OA and T2DM across multiple domains of the TDF. This study is an important step towards developing an intervention to improve diagnosis and evidence-based treatment of knee OA in persons with T2DM.

## Author contributions

LKK and GAH conceived of the study. LKK, OK, EJW, CM, IS, JS, AW, NMI, LL, GAH, and JAP contributed to the study design. LKK and EJW contributed to data collection. LKK, OK, EJW, CM, IS, GAH, and JAP were involved in data analysis. LKK drafted the article with assistance from OK. All authors critically revised the article and approved the final version for submission. All authors had full access to all the data in the study. LKK, EJW, CM, IS, GAH directly accessed and verified the underlying data reported in the Article.

## Role of the funding source

This study was funded by a Project Grant, PJT-166069, from the Canadian Institutes of Health Research (CIHR). The funder had no role in the design and conduct of the study; collection, management, analysis, and interpretation of the data; preparation, review, or approval of the manuscript; or the decision to submit the manuscript for publication.

## Data statement

The qualitative data generated and/or analyzed during the current study are not publicly available due to the Research Ethics Board-approved study protocol. However, the data can be made available from the corresponding author on reasonable request.

## Declaration of competing interest

GAH has received research support as the Sir John and Lady Eaton Professor and Chair of Medicine, Department of Medicine, University of Toronto; LL receives salary support as the Director of the Novo Nordisk Network for Healthy Populations, University of Toronto; all other authors declare no other relationships or activities that could appear to have influenced the submitted work.
